# Carotenoid Raman Signatures are Better Preserved in Dried Cells of the Desert Cyanobacterium *Chroococcidiopsis* than in Hydrated Counterparts after High-Dose Gamma Irradiation

**DOI:** 10.3390/life10060083

**Published:** 2020-06-08

**Authors:** Mickael Baqué, Alessandro Napoli, Fagliarone Claudia, Ralf Moeller, Jean-Pierre de Vera, Daniela Billi

**Affiliations:** 1 German Aerospace Center (DLR), Institute of Planetary Research, Department of Planetary Laboratories, Astrobiological Laboratories, 12489 Berlin, Germany; Mickael.Baque@dlr.de (M.B.); jean-pierre.devera@dlr.de (J.-P.d.V.); 2 Department of Biology, Laboratory of Astrobiology and Molecular Biology of Cyanobacteria, University of Rome Tor Vergata, 00133 Rome, Italy; alessandro.napoli@uniroma2.it (A.N.); claudia.fagliarone@hotmail.it (C.F.); 3 Space Microbiology Research Group, Radiation Biology Department, Institute of Aerospace Medicine, German Aerospace Center (DLR), 51147 Cologne, Germany; Ralf.Moeller@dlr.de

**Keywords:** biosignatures, extremophiles, ionizing radiation, Raman spectroscopy, cyanobacteria, Mars exploration

## Abstract

Carotenoids are promising targets in our quest to search for life on Mars due to their biogenic origin and easy detection by Raman spectroscopy, especially with a 532 nm excitation thanks to resonance effects. Ionizing radiations reaching the surface and subsurface of Mars are however detrimental for the long-term preservation of biomolecules. We show here that desiccation can protect carotenoid Raman signatures in the desert cyanobacterium *Chroococcidiopsis* sp. CCMEE 029 even after high-dose gamma irradiation. Indeed, while the height of the carotenoids Raman peaks was considerably reduced in hydrated cells exposed to gamma irradiation, it remained stable in dried cells irradiated with the highest tested dose of 113 kGy of gamma rays, losing only 15–20% of its non-irradiated intensity. Interestingly, even though the carotenoid Raman signal of hydrated cells lost 90% of its non-irradiated intensity, it was still detectable after exposure to 113 kGy of gamma rays. These results add insights into the preservation potential and detectability limit of carotenoid-like molecules on Mars over a prolonged period of time and are crucial in supporting future missions carrying Raman spectrometers to Mars’ surface.

## 1. Introduction

The surface of Mars is a hostile environment for the preservation of signatures of past life due to the presence of oxidizing species, mainly perchlorates [1], and ionizing radiation [2] that can degrade organic molecules directly or through secondary oxidants [3]. Polymers that store and transfer information for life, namely DNA, RNA, and proteins, being extremely unlikely to be synthesized abiotically in long chains, are considered the most direct signs of extant or extinct life [4]. Indeed the detection of nucleic acids or peptides, with lengths in the range of tenths of monomers, is considered difficult to be refuted as a successful life detection [5]. In addition, pigments, being the product of specific biochemical pathways, are considered unambiguously of biogenic origin, and, among them, carotenoids are promising targets since synthetized by phototrophic and non-phototrophic microorganisms [6,7].

Carotenoids have been included in a high priority target list for biomolecule detection on Mars [8]. Remarkably these molecules are generally easy to detect by Raman spectroscopy, and this a relevant feature since ESA/Roscomos’ ExoMars Rosalind Franklin rover and NASA’s Perseverance rover will carry Raman spectrometers, among other instruments, to detect signatures of life on Mars [9,10,11]. 

To prepare for and support these missions, a deeper understanding of life resilience and of its constituents under extraterrestrial stresses is of primordial importance. Ground-based and space exposure experiments are complementary in this approach [12]. They have shown the extreme hardiness of an important variety of organisms, pushing the limits of life as we know it, and testing potential targets for future search-for-life missions to our Solar System [12,13]. 

Biomarker stability/degradation have, for instance, been evaluated in desert cyanobacteria of the genus *Chroococcidiopsis* exposed to different types of ionizing radiation [14] and Mars conditions simulated by using the EXPOSE facility installed outside the International Space Station [15]. 

Although UV radiation exposure is considered the most destructive aspect of exposure to space conditions [16], it penetrates only millimeters into regolith and is therefore easily shielded against it [17]. When dried *Chroococcidiopsis* cells mixed with Martian mineral analogues were exposed to 570 MJ/m^2^ of UV radiation (200–400 nm), corresponding to half a Martian year, the β-carotene Raman signal was still detectable with Raman spectroscopy, although reduced to 2% of control [18]. Photosynthetic pigments also resulted strongly bleached when analyzed by confocal laser scanning microscopy, even though autofluorescence still occurred in a few cells in thicker layers, while DNA was detectable by polymerase chain reaction, only in cells mixed with minerals [18]. These investigations showed that Martian mineral analogues provide protection against the effect of UV radiation. 

During the EXPOSE-E and EXPOSE-R space missions, the exposure in low Earth orbit resulted in the failure in detecting β-carotene in epilithic cyanobacterial biofilms [19] and in *Chroococcidiopsis* cells layered on rocks [20]. 

During the EXPOSE-R2 space mission, in the contest of the BIOlogy and Mars Experiment (BIOMEX), dried cyanobacteria were mixed with Martian mineral analogues to mimic Martian subsurface radiation conditions reached by a reduced amount of UV radiation [21], such as it could be the case within potential subsurface or endolithic habitable niches. Dried *Chroococcidiopsis* cells mixed with Martian mineral analogues were exposed to a total dose of 2.19 × 10^2^ kJ/m and 1.94 × 10^2^ kJ/m UV radiation (200–400 nm), depending on the position inside the facility and presence of a 0.1% neutral filter [15], and 0.5 Gy of cosmic ionizing radiation [22]. Exposed cells with minerals showed a reduction in the photosynthetic pigments autofluorescence to about 20% of control while DNA could not be amplified in exposed cells not mixed with minerals [15].

On the other hand, ionizing radiation penetrates meters into regolith thus causing structural and chemical changes on organic and biological molecules [23]. However, the radiation environment in low Earth orbit is not fully representative of Mars due to the Earth’s magnetic field shielding high-energy charged particles [12]. Thus, exposure campaigns to high doses of different types of ionizing radiation (X-rays, gamma-rays, heavy ions) were performed in the frame of the STARLIFE project [24]. After exposure to a gamma irradiation dose of 117 kGy, desiccated algae (photobiont of the lichen *Circinaria gyrosa*) showed a detectable carotenoid Raman signal, although altered [25]. While in dried cells of the cyanobacterium *Nostoc* sp. strain CCCryo 231-06 the carotenoid signal was lost after exposure to 27 kGy of gamma irradiation, but it was still detectable in cells mixed with Martian regolith simulants irradiated with 117 kGy of gamma rays [26]. After exposure to 113.25 kGy of gamma rays dried, *Chroococcidiopsis* cells not mixed with minerals possessed detectable DNA and photosynthetic pigments [14]. 

If life ever arose on Mars as its surface conditions deteriorated, it could have found refuge in subterranean environments with “punctuated” habitability due to transient availability of liquid water [27]. Since this fluctuation might have been experienced by remnants of putative past life, it is crucial to investigate the preservation potential of biomarkers subjected to ionizing radiation in presence or absence of liquid water.

In this work, we investigated by confocal Raman spectroscopy the permanence of the carotenoid signal in a desert strain of *Chroococcidiopsis* in two experimental conditions: one-year of air-dried storage, and exposure of hydrated and dried cells to increasing doses of γ-rays up to 113.25 kGy. 

## 2. Materials and Methods

### 2.1. Cyanobacterial Strain and Culture Conditions

*Chroococcidiopsis* sp. CCMEE 029 was isolated by Roseli Ocampo-Friedmann from cryptoendolithic growth in sandstone from Negev Desert (Israel). The strain is maintained at the Laboratory of Astrobiology and Molecular Biology of Cyanobacteria, Department of Biology, University of Rome Tor Vergata, as part of the Culture Collection of Microorganisms from Extreme Environments (CCMEE) established by E. Imre Friedmann and Roseli Ocampo-Friedmann. The cyanobacterial strain was grown in BG11 medium by using 50-mL vented flasks, inside an incubator at 25 °C, without shaking, under a photon flux density of 40 μmol m^−2^ s^−1^ provided by cool-white fluorescent lamp (4100 K) with a 16 h/8 h light/dark cycle. 

### 2.2. Sample Preparation and Irradiation

Samples containing approximately 1.5 × 10^8^ cells were prepared by using 2-month liquid cultures in the early stationary phase. Dried samples were prepared by filtering cell aliquots on Millipore filters and air-drying overnight in a sterile hood. Liquid samples were prepared in 50 µL of BG11. 

Samples were stored inside Eppendorf-safe lock tubes and irradiated with increasing doses of low Linear Energy Transfer (LET) γ-rays in July 2015 during the STARLIFE irradiation campaign, Table 1 [14,24]. Gamma irradiation was generated using a C-188 cobalt-60 source provided by Beta-Gamma-Service (BGS) in Wiehl, Germany, with a dose rate of 100 Gy/min (and a LET value of 0.2 keV/µm from secondary electrons), at room temperature, in the dark. After irradiation, dried cells immobilized on filters were kept inside Eppendorf-safe-lock tubes and stored in the dark, at room temperature, in oxygenic atmosphere; while hydrated cells were shipped on ice and analyzed immediately after the irradiation campaign.

### 2.3. Raman Spectroscopy: Set-up and Spectra Parameters

**Figure 1:**
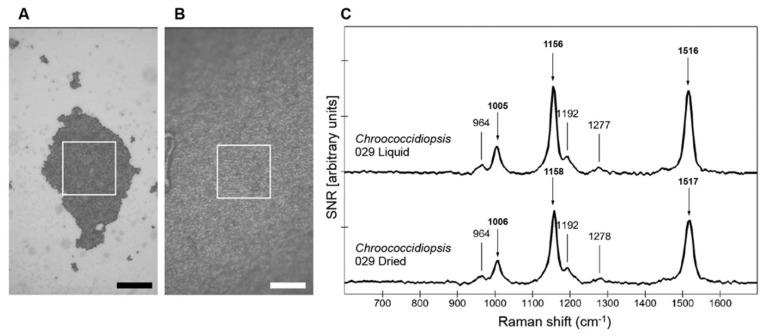
Representative image scans (white squares) of *Chroococcidiopsis* sp. CCMEE 029: hydrated cells (**A**) and dried cells (**B**). Stacked spectra of hydrated and dried (**C**) with a signal-to-noise ratio (SNR) > 90% of the maximum recorded for each sample. Both spectra are represented with the same scale (1s integration time, 1 accumulation, and 1 mW applied laser power). Main peaks’ positions are indicated in cm^−1^. Bar = 100 μm.

Raman measurements were performed with a confocal WITec alpha300 Raman microscope operating at room temperature, under ambient atmospheric conditions. The Raman laser excitation wavelength was 532 nm and the spectral resolution of the spectrometer 4–5 cm^−1^. A Nikon 10× objective, with a 0.25 numerical aperture, was used to focus the laser on a 1.5 µm spot. The surface laser power was set at 1 mW. A spectral calibration was performed with a pure silicon test sample. Spectra were acquired directly on fragments of about 2 mm^2^ of the Millipore filters for the dried samples and on 10 µL air-dried drops on a microscopy glass slide for the liquid samples. Acquisition time was kept between 0.5 and 1 s to avoid signal saturation from photosynthetic pigments’ fluorescence with 1 accumulation. Single spectra, line scans, and image scans with up to 150 μm × 150 μm and up to 2500 image points (see for example Figure 1) were obtained thus collecting a minimum of 2500 measurements per sample. 

### 2.4. Data Processing

In order to compare the different irradiation effects on the cyanobacteria samples’ spectra, exposed in dried or liquid forms, we chose two parameters: the absence/presence of signal, defined as the signal coverage, and the value of the signal-to-noise ratios (SNR), as described previously [26]. 

The spectra were treated with the WITec Project FIVE for data analysis. First, by cropping to the region of interest (between 600 and 1700 cm^−1^), then by applying a fourth-order polynomial function for background subtraction and finally by creating SNR masks. We defined the SNR for the carotenoids’ spectra as the height of the 1515 cm^−1^ peak divided by the noise represented as the standard deviation of the spectral region near the Raman peaks (700 to 900 cm^−1^). For further investigation, the spectra of each image scan having an SNR superior or equal to 90% of the maximal value were averaged. The peak heights of all peaks (expressed in SNR values) were calculated from the resulting averaged spectrum by using Lorentzian fits and were normalized to the corresponding SNR of the non-irradiated sample (control). The signal coverage was derived from a SNR intensity mask selecting spectra presenting only a SNR superior to 4 for a minimum of 2500 spectra per sample.

### 2.5. Bioinformatics Analysis 

Genes encoding enzymes of the carotenogenesis biosynthesis pathways were identified in the genome of *Chroococcidiopsis* sp. CCMEE 029 by using BlastKOALA [28]. The genome sequenced was obtained by using Illumina/Solexa technology (CD Genomics, Shirley, NY, USA), and a draft genome sequence was annotated by using the software tool PROKKA v.1.11 [29] and the interface provided by the Galaxy-based framework Orione [30]. 

## 3. Results

### 3.1. Comparison of Carotenoid Raman Signal in Dried and Hydrated Cells 

The effect of desiccation on the Raman spectra of *Chroococcidiopsis* sp. CCMEE 029 was determined according to the value of the signal-to-noise ratios (SNR) of hydrated and dried cells’ spectra. Treating the acquired spectra in signal-to-noise ratios and using different masks, to select sub-populations of spectra, allowed the comparison between the two different samples independently to inhomogeneity of the area chosen for the image scans (Figure 1A,B). 

The stacked spectra of both hydrated cells and dried cells of strain CMEE 029 showed a typical Raman signal as expected for cyanobacteria [31], consisting of three main carotenoid peaks. Hydrated cells showed two strong peaks at ~1516 and ~1156 cm^−1^, characteristic of the C=C and C–C stretching vibrations, and a weaker feature at ~1005 cm^−1^, which corresponds to the rocking modes of the CH_3_ groups (Figure 1C top). These peaks were evident also in dried cells, although only slightly reduced in intensity. Compared to the SNR values of hydrated cells, dried cells showed lower values: 130 versus 100 SNR for the 1516 cm^−1^ peak (Figure 1C bottom).

### 3.2. Degradation of Carotenoid Raman Signatures in Hydrated Cells 

**Figure 2:**
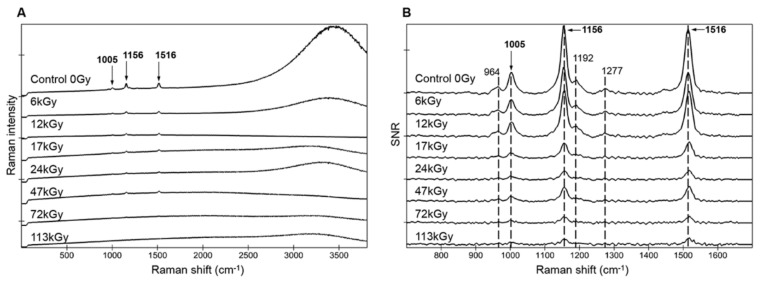
Stacked spectra for hydrated *Chroococcidiopsis* sp. CCMEE 029 exposed to increasing gamma ray doses (Control 0 Gy, irradiated cells from 6 kGy to 113 kGy). Average spectra without correction from image scans, > 2500 spectra (**A**). Spectra with a signal-to-noise ratio (SNR) > 90% of the maximum recorded for each sample and approximate peak positions (**B**). All spectra are represented with the same scale (1s integration time, 1 accumulation, and 1 mW applied laser power).

Hydrated cells of strain CCMEE 029 were exposed to increasing doses of gamma irradiation and analyzed by Raman mapping and single points (Figure 2A,B). The uncorrected spectrum of hydrated control cells (0 Gy) presented the typical carotenoids signatures (three main peaks at 1005, 1156, and 1516 cm^−1^) and a strong fluorescence at about 3300 cm^−1^ (650 nm), due to phycocyanin, allophycocyanin, and chlorophyll *a* (Figure 2A). The fluorescence signal of photosynthetic pigments and the Raman signal of carotenoids resulted more disturbed with increasing radiation doses and they became hardly apparent after the dose of 47 kGy (Figure 2A).

Then spectra showing the maximal signal, hence more representative than average spectra with different sampling zones were extracted for each sample by a mask selecting the spectra with SNR values superior to 90% of the maximum recorded one. Selecting and comparing only a single maximum spectrum for each sample (out of 2500 spectra) would result in biases inherent to the heterogeneities due to sample preparation, irradiation, and spectra acquisition. The spectra with a SNR > 90% of the maximum recorded for each sample showed evident signal degradation with increasing doses. The heights of the three carotenoid peaks dropped significantly after 17 kGy. While the weaker carotenoid features, at 964, 1192, and 1278 cm^−1^, were still present up until 17 kGy, only the three main peaks (1005, 1156, and 1516 cm^−1^) remained visible up to the highest dose of 113 kGy (Figure 2B). The peaks’ positions, taken on the complete image scans (>2500 spectra), did not change significantly with increasing radiation doses with less than 0.1% variability: 1004.6 ± 0.8, 1155.9 ± 0.9, and 1515.4 ± 0.8 cm^−1^.

### 3.3. Preservation of Carotenoid Raman Signal in Dried Cells 

**Figure 3:**
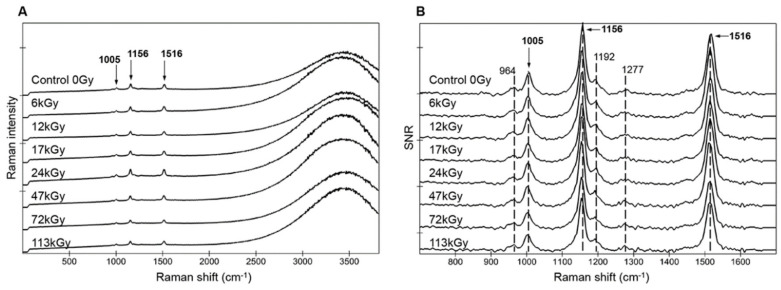
Stacked spectra for dried *Chroococcidiopsis* sp. CCMEE 029 exposed to increasing gamma ray doses (Control 0 Gy, irradiated cells from 6 kGy to 113 kGy). Average spectra without correction from image scans, > 2500 spectra (**A**). Spectra with a signal-to-noise ratio (SNR) > 90% of the maximum recorded for each sample and approximate peak positions (**B**). All spectra are represented with the same scale (1s integration time, 1 accumulation, and 1 mW applied laser power).

Spectra of dried cells of strain CCMEE 029 also showed the typical three carotenoid peaks and the fluorescence signal of photosynthetic pigments (Figure 3A). After exposure to increasing doses of ionizing radiation, the fluorescence signal of photosynthetic pigments and the three carotenoid peaks resulted equally preserved after exposure up to the highest dose of 113 kGy (Figure 3A).

The spectra with signal-to-noise ratio (SNR) > 90% showed that carotenoids’ Raman signatures remained mostly unchanged for dried cells as the three main carotenoid peaks (1005, 1156, and 1516 cm^−1^), as well as the weaker features (964, 119C2, and 1278 cm^−1^), remained undisturbed up until the highest dose of 113 kGy. Similar to the hydrated samples, the peaks’ positions, taken on the complete image scans (>2500 spectra), showed no significant variation with increasing radiation doses with less than 0.1% variability: 1003.6 ± 0.4, 1154.9 ± 0.7, and 1514.9 ± 0.8 cm^−1^.

A mask selecting only spectra with a SNR > 4 allowed the calculation of the coverage of the signal, providing an indication of the potential degradation (Table 2). Values were more homogeneous for dried cells with a constant full coverage of the signal (100%). While hydrated cells showed a less homogeneous signal coverage according to the dose (Table 2), probably due to the area chosen for image scans (Figure 1). 

**Figure 4:**
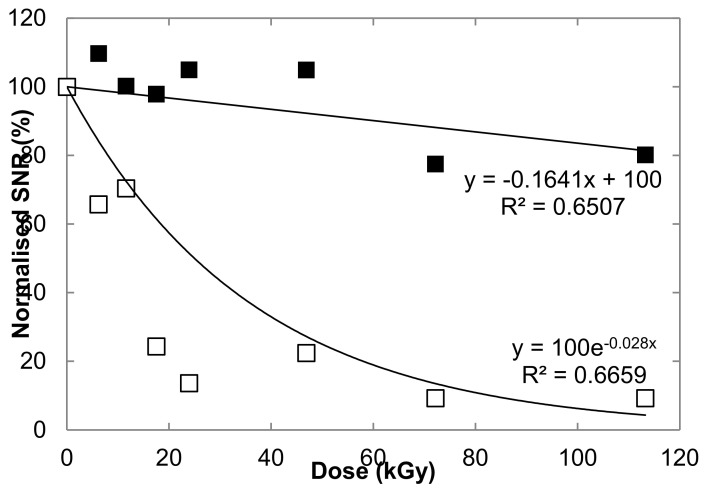
Intensity of the 1516 cm^−1^ peak expressed in SNR normalized to the respective non-irradiated controls (hydrated or dried) versus gamma ray doses (kGy) of strain CCMEE 029 exposed as hydrated cells (empty squares) or dried cells (filled squares).

The peak heights of the carotenoid signal, expressed in SNR values normalized to the non-irradiated controls, clearly showed the difference between hydrated and dried cells (Figure 4). The 1516-cm^-1^-carotenoid-peak intensity of dried cells exposed to 113 kGy retained 80% of the non-irradiated control value, while for hydrated cells it dropped to 9% of the control (Figure 4). No clear trend was observed according to the dose for the dried samples. For hydrated cells, the intensity of the 1516 cm^−1^ peak showed a potential exponential decay according to the dose (Figure 4), but the values for both 72 kGy and 113 kGy irradiation doses were very similar (9.24% and 9.25%, respectively). All three main carotenoid peaks (1005, 1156, and 1516 cm^−1^) followed the same trend in all samples (not shown) but the 1516-cm^−1^ one strongly correlated to carotenoid concentrations (see e.g., [32,33]). 

### 3.4. Identification of Carotenogenesis Genes 

The *in silico* analysis of *Chroococcidiopsis* sp. CCMEE 029′s genome determined the presence of putative genes encoding enzymes of carotenogenesis biosynthesis pathways (Table 3). The identified genes were: (i) genes encoding enzymes for the early steps of carotenoid biosynthesis, including geranylgeranyl phytoene synthase (CrtB), 15-cis-phytoene desaturase (CrtP), zeta-carotene desaturase (CrtQ), cis-carotene isomerase (CrtH), phytoene desaturase (CtrI); (ii) one gene for the synthesis of the hydroxylated carotenoid zeaxanthin (CrtR); (iii) genes encoding two distinct carotene ketolases for the synthesis of the keto-carotenoid canthaxanthin and echinone (CrtO and/or CrtW) and of the carotenoid glycoside 4-ketomyxol 2′-fucoside (CrtW). Putative genes encoding enzymes CruF and CruG for the conversion of γ-carotene to myxol 2′-methylpentoside were absent. The sequence similarity of the newly determined *Chroococcidiopsis* sequences showed the highest homology with heterocystous cyanobacteria (Table 3). 

## 4. Discussion

Here we investigated by confocal Raman spectroscopy the preservation of photosynthetic pigments and carotenoids in a desert strain of the cyanobacterium *Chroococcidiopsis* exposed to desiccation and increasing doses of γ-ray radiation that were carried out in the context of the STARLIFE project [14,24]. The Raman spectra at 532-nm excitation of hydrated cells showed an important fluorescence signal at ~3300 cm^−1^ (~650 nm) due to photosynthetic pigments, mainly phycobiliproteins, phycocyanin and allophycocyanin, and chlorophyll *a* [18]. In addition, thanks to the resonance effects with a 532-nm excitation wavelength, a typical carotenoid signal with three main peaks at 1005, 1156, and 1516 cm^−1^ was clearly present. 

Desiccation slightly reduced the intensity of the carotenoid peaks after 1-year of air-dried storage. The fluorescence signal of photosynthetic pigments was also slightly reduced after desiccation. The scored pigment permanence might be ascribable to the capability of desert cyanobacterium to stabilize its sub-cellular components by accumulating trehalose and sucrose along with its capability of coping with oxidative stress. Indeed, strain CCMEE 029 has been reported to accumulate trehalose and sucrose in response to water stress [34], and, thanks to an efficient antioxidant defense system, to avoid protein oxidative damage when exposed to 0.5 M hydrogen peroxide, 1-year desiccation, and 25 kGy of γ-rays [35]. 

When hydrated cells of strain CCMEE 029 were exposed to increasing doses of γ-rays, the signal of photosynthetic pigments and carotenoids was more disturbed with increased radiation doses and was almost undetectable on average spectra after exposure to 47 kGy. However, when spectra showing the maximal signal for hydrated cells were considered (with a SNR > 90% of the maximum), the three main peaks of carotenoids (1005, 1156, and 1516 cm^−1^) were still detectable after 113 kGy. The detectability of the carotenoid signal in hydrated *Chroococcidiopsis* cells exposed to 113 kGy is remarkable when compared with that of the cyanobacterium *Synechocystis* sp. PCC 6803. In the latter, the carotenoid signal was reduced to 66–72% of the control after 15 kGy of γ-irradiation and was totally destroyed after 150 kGy [36]. On the other hand, in hydrated *Chroococcidiopsis* cells exposed to 113 kGy of γ-irradiation, the carotenoid signal was still present, although reduce to 9% of control. Moreover, the signal coverage (Table 2) showed that this value was not due to a few protected cells as it covered the entire scanned areas. The observed difference between hydrated, irradiated cyanobacteria might be due to the oxidative-stress sensitivity of *Synechocystis* sp. PCC 6803 compared to the oxidative-stress tolerance of *Chroococcidiopsis* sp. CCMEE 029. Indeed, unlike *Chroococcidiopsis*, *Synechocystis* underwent extensive protein oxidative damage when exposed to hydrogen peroxide, desiccation, and γ-irradiation [35]. Since, it was also reported that hydrated cells of *Chroococcidiopsis* sp. CCMEE 029 died when exposed to 24 kGy of γ-rays [14], this suggests that despite cell death, carotenoids have not undergone complete degradation. 

The composition and structure of carotenoids occurring in different organisms is also relevant. For instance, the Raman signal of the desiccation-, radiation-tolerant bacterium *Deinococcus radiodurans* exposed to γ-rays, showed a carotenoid signal that was deeply altered after 15 kGy and completely degraded after 150 kGy [36]. Deinoxanthin, the dominant carotenoid synthesized by *D. radiodurans*, is a longer chain molecule than β-carotene (it has eleven conjugated bonds in its polyene backbone compared to nine bonds in β-carotene). In addition, by having only one terminal ring structure, deinoxanthin is more prone to radiolytic breakdown, thus resulting in an efficient reactive oxygen species (ROS) scavenger, and hence more rapidly degraded upon irradiation [36]. 

Although the exact pigment composition of *Chroococcidiopsis* sp. CCMEE 029 is unknown, the bioinformatics analysis of its genome revealed the presence of genes encoding enzymes for the biosynthesis of carotenoids (β-carotene), of hydroxylated carotenoids (zeaxanthin), of keto-carotenoids (canthaxanthin and echinone), and of carotenoid glycosides (4-ketomyxol 2′-fucoside).

β-carotene, canthaxanthin, echinone, and β-cryptoxanthin have nine conjugated bonds in their polyene backbone compared to eleven for 4-ketomyxol 2′-fucoside and eight for zeaxanthin. The peak corresponding to the C=C stretching vibrations is a strong indicator of the number of conjugative bonds in carotenoid and xanthophyll molecules: The higher the number of conjugated bonds the lower its wavenumber [36]. For example, the Raman spectra of *D. radiodurans* clearly showed the dominant presence of the xanthophyll deinoxanthin with eleven conjugated bonds and a ν_1_(C=C) peak at around 1510 cm^−1^ [32,36]. Cyanobacteria contain principally β-carotene with a ν_1_(C=C) peak at higher wavenumber around 1516–1518 cm^−1^ [32,36,37]. This observation is in accordance with our results on *Chroococcidiopsis* showing a 1516 cm^−1^ ν_1_(C=C) peak and therefore the dominance of β-carotene and other similarly structured carotenoids. Interestingly however, the presence in this cyanobacterium of several carotenoid biosynthesis pathways indicated the presence of an efficient ROS scavenging system. This antioxidant system should have the high peroxy radicals inactivation capacities of xanthophylls, and the effective quenching capacities and high resistance towards peroxy radicals of keto-derivatives [38]. On the other hand, glycosylated carotenoid derivatives (ketomyxoxanthophylls, like 4-ketomyxol 2′-fucoside) have been shown to participate actively in the structural stabilization of cyanobacterial membranes and to play a role also as antioxidants [39,40]. Our Raman data did not show any variation in the carotenoids’ peak positions with increasing radiation damage, as it would be expected if for example, 4-ketomyxol 2′-fucoside was abundant in *Chroococcidiopsis* and preferentially degraded by ROS-mediated damage than its shorter-chain counterparts. In endolithic cyanobacterial communities, dominated by *Chroococcidiopsis*-like cells, a depth-dependent shift towards lower wavenumbers with increasing proximity to the rock surface has been reported, indicating the presence of longer chain carotenoids, that was interpreted as a response towards increased light intensity and oxidative stress [41]. In the present study, the highest dose of 113 kGy was reached in about 19 hours of irradiation in the dark. However, the investigated strain might have exploited a light-independent energy generation pathway, as reported for *Chroococcidiopsis* cells in subsurface rock samples [42]. Therefore an in-depth carotenoid characterization in hydrated, irradiated cells should be performed to get insights on their synthesis in response to ionizing radiation.

On the other hand, the Raman analysis revealed a greater stability of photosynthetic pigments and carotenoids in dried *Chroococcidiopsis* cells exposed to increasing doses of γ-rays. Indeed all the spectra showed three main carotenoid peaks (1005, 1156, and 1516 cm^−1^) and weaker ones (964, 1192, and 1278 cm^−1^) up to the highest dose of 113 kGy. Hence dried *Chroococcidiopsis* cells were characterized by an enhanced detectability of photosynthetic pigments and carotenoids compared to dried cells of *Nostoc* sp. CCCryo 231-06 that were irradiated in the context of the same STARLIFE campaign [26]. In dried *Nostoc* cells the carotenoid signal was visible until exposure to 27 kGy of γ-ray irradiation, while at 56 kGy it was present only in 0.2% of the spectra and completely lost at 81 kGy and 117 kGy [26]. On the contrary, the carotenoid signal was preserved in dried *Nostoc* cells when embedded in Martian mineral analogues and exposed to 117 kGy of γ-rays [26]. It was hypothesized that in this cyanobacterium, the moisture absorption and retention capacities associated to its desiccation tolerance might have been detrimental to biomarker preservation under high doses of ionizing radiations [26]. Therefore, the degradation of carotenoids during irradiation might have been a consequence of indirect mechanisms via free radical production rather than direct radiation damage.

It has been reported that dried cells of *Chroococcidiopsis* sp. CCMEE 029 were killed by 47 kGy of γ-rays [14]. Nevertheless, the Raman spectra were preserved after a dose as high as 113 kGy followed by 1-year of air-dry storage. This result is in line with the carotenoid Raman signal, still detectable, although disturbed, in dried photobiont of the lichen *Circinaria gyrosa*, irradiated with 117 kGy of γ-rays, during the same STARLIFE campaign [25].

In the present study the hydration state of *Chroococcidiopsis* cells resulted to be the most detrimental factor for the preservation of the carotenoid Raman signal after ionizing radiation, and this observation is in line with previous experiments. Metabolically inactive and dormant organisms, namely anhydrobiotes, capable of stabilizing their sub-cellular components when dried [43], are expected to be much less affected by indirect radiation damage and their biomolecules could be potentially preserved for thousands to millions of years. Indeed ancient and fossil carotenoids have been detected in ancient halite brine inclusions (9 ka to 1.44 M in age) by Raman spectroscopy [44] and their diagenetic products (e.g., β-carotane) in 1.64 billion-year old samples by Gas-chromatography Mass spectroscopy [45] in different settings sharing their lack of oxygen during burial thus protected from oxidizing conditions and ROS. 

The presence of mostly unchanged carotenoid signal in dried *Chroococcidiopsis* cells irradiated with increasing γ-ray doses as well as its detectability, although with reduced intensity, in hydrated cells irradiated with 113 kGy, added new insights into the preservation potential and detectability limit of carotenoid-like molecules on Mars, over a prolonged period of time. Moreover, previous findings showed the protection provided by Martian mineral analogues to the Raman carotenoid signal of dried cells of *Nostoc* sp. CCCryo 231-06 irradiated with 117 kGy of γ-rays [26].

NASA’s Curiosity rover measured an ionizing radiation dose of 76 mGy/year at the Gale Crater’s surface and estimated a dose of 8.7 mGy/year at 2 m depth [2]. Thus, we can estimate the order of magnitude provided here by 113 kGy of γ-rays to correspond roughly to about 1.5 million years on the Martian surface and 13 million years at 2 m depth. The ExoMars Rosalind Franklin rover, by drilling below 2 m of the Martian surface, and by operating a Raman spectrometer with a 532-nm laser, could potentially detect ancient carotenoid-like signatures preserved for millions of years. Furthermore, temperature is known to influence radiation-induced damage, low temperatures minimizing indirect damages due to a reduced ROS mobility [46]. Hence Mars, being extremely dry and cold, has features relevant to biomarker preservation, despite the lack of a magnetic field and of a thick atmosphere providing protection against solar and cosmic ionizing radiations.

The permanence of the carotenoid signal in hydrated, irradiated *Chroococcidiopsis* should be further investigated in ionizing-irradiation campaigns at low temperatures under Mars-like salty environments, e.g., saline or perchlorate-rich conditions. Indeed the possible preservation of biological information in chloride-rich and perchlorate materials over geologic timescales has been reported and salt-bearing materials have been suggested to be high-priority targets for searching evidence for life on Mars [47]. Moreover, an in-depth characterization of the carotenoid content in metabolically active *Chroococcidiopsis* cells under Mars-like conditions might be relevant to further characterize changes in pigment composition under extraterrestrial conditions.

## Figures and Tables

**Table 1:** γ-ray doses in kGy, Linear Energy Transfer (LET) 0.2 keV/µm as performed during the STARLIFE irradiation campaign.

Dose (Nominal)	Dose (Applied)	Exposure Time
0	0	0
6	6.21	1 h
12	11.59	2 h
18	17.51	3 h
24	23.92	4 h
47	46.88	7 h 50′
72	72.16	12 h
113	113.25	18 h 50′

**Table 2:** γ-rays doses description (Cobalt 60 gamma irradiation, nominal values in kGy) and calculated signal coverage with SNR > 4 filter (in%) on n ≥ 2500 spectra.

Nominal Dose	*Chroococcidiopsis* sp. CCMEE 029 Dried	*Chroococcidiopsis* sp. CCMEE 029 Liquid
0	100.0%	100.0%
6	100.0%	100.0%
12	100.0%	96.4%
18	100.0%	100.0%
24	100.0%	99.5%
47	100.0%	99.8%
72	100.0%	73.7%
113	100.0%	100.0%

**Table 3:** Carotenogenesis genes in *Chroococcidiopsis* sp. CCMEE 029.

Gene	KEGG Enzyme	Closest BlastN Hit (GenBank ID)	Identity %	Genbank Accession n°
*crtQ*	K00514	*Nostoc* sp. cyanobiont (CP026692.1)	79.69%	MT438481
*crtB*	K02291	*Scytonema* sp. HK-05 (AP018194.1)	76.91%	MT438479
*crtO(1)*	K02292	*Scytonema* sp. HK-05 (AP018194.1)	82.29%	MT438474
*crtO(2)*	K02292	*Scytonema* sp. HK-05 (AP018194.1)	80.32%	MT438476
*crtP*	K02293	*Tolypothrix* sp. PCC 7910 (CP050440.1)	79.44%	MT438478
*crtR*	K02294	*Scytonema* sp. HK-05 (AP018194.1)	78.79%	MT438480
*crtH*	K09835	*Crinalium epipsammum* PCC 9333 (CP003620.1)	77.51	MT438471
*crtW*	K09836	*Nostoc* sp. ATCC 53,789 (CP046703.1)	73.80%	MT438477
*crtU*	K09879	*Calothrix* sp. NIES-2100 (AP018178.1)	72.65%	MT438469
*crtI(1)*	K10027	*Gloeobacter kilaueensis* JS1 (CP003587.1)	72.02%	MT438468
*crtI(2)*	K10027	*Nostoc linckia* NIES-25 (AP018223.1)	71.80%	MT438473
*cruA*	K14605	*Calothrix* sp. PCC 7507 (CP003943.1)	75.25%	MT438470
*cruP*	K14606	*Nostoc* sp. NIES-4103 (AP018289.1)	77.59%	MT438467
*crtX*	K14596	*Scytonema* sp. NIES-4073 (AP018268.1)	78.09%	MT438475
*crtY*	K22502	*Calothrix* sp. NIES-4105 (AP018290.1)	72.97%	MT438472
